# French hepatitis C care cascade: substantial impact of direct-acting antivirals, but the road to elimination is still long

**DOI:** 10.1186/s12879-020-05478-6

**Published:** 2020-10-15

**Authors:** Cécile Brouard, Josiane Pillonel, Marjorie Boussac, Victor de Lédinghen, Antoine Rachas, Christine Silvain, Nathalie Lydié, Stéphane Chevaliez, Corinne Pioche, Julien Durand, Florence Lot, Elisabeth Delarocque-Astagneau

**Affiliations:** 1 Santé publique France, the National Public Health Agency, Saint-Maurice, France; 2 French National Health Insurance (Cnam), Paris, France; 3 Haut-Lévêque Hospital, Bordeaux University Hospital, Pessac, France; 4 Inserm U1053, Bordeaux University, Bordeaux, France; 5 Hepatology Unit, University Hospital, Poitiers, France; 6 National Reference Centre for Viral Hepatitis B, C and Delta, Henri Mondor University Hospital, Créteil, France; 7 Inserm U955, Paris-Est University, Créteil, France; 8 Paris-Saclay University, Versailles Saint-Quentin University, Inserm, CESP, Anti-infective evasion and pharmacoepidemiology, Villejuif, France; 9 AP-HP (Public Assistance-Paris Hospitals), Paris-Saclay University hospital group, Raymond-Poincaré Hospital, Hospital Department of Epidemiology and Public Health, Garches, France

**Keywords:** Hepatitis C, Cascade of care, Elimination, Direct-acting antivirals, Prevalence, Diagnosis, Management, Treatment, France

## Abstract

**Background:**

Hepatitis C virus (HCV) elimination by 2030, as targeted by the World Health Organization (WHO), requires that 90% of people with chronic infection be diagnosed and 80% treated. We estimated the cascade of care (CoC) for chronic HCV infection in mainland France in 2011 and 2016, before and after the introduction of direct-acting antivirals (DAAs).

**Methods:**

The numbers of people (1) with chronic HCV infection, (2) aware of their infection, (3) receiving care for HCV and (4) on antiviral treatment, were estimated for 2011 and 2016. Estimates for 1) and 2) were based on modelling studies for 2011 and on a virological sub-study nested in a national cross-sectional survey among the general population for 2016. Estimates for 3) and 4) were made using the National Health Data System.

**Results:**

Between 2011 and 2016, the number of people with chronic HCV infection decreased by 31%, from 192,700 (95% Credibility interval: 150,900-246,100) to 133,500 (95% Confidence interval: 56,900-312,600). The proportion of people aware of their infection rose from 57.7 to 80.6%. The number of people receiving care for HCV increased by 22.5% (representing 25.7% of those infected in 2016), while the number of people on treatment increased by 24.6% (representing 12.1% of those infected in 2016).

**Conclusions:**

This study suggests that DAAs substantially impact CoC. However, access to care and treatment for infected people remained insufficient in 2016. Updating CoC estimates will help to assess the impact of new measures implemented since 2016 as part of the goal to eliminate HCV.

## Introduction

With the advent of highly effective direct-acting antivirals (DAAs) for hepatitis C virus (HCV) infection, in 2014, the World Health Assembly pledged to eliminate HCV infection as a public health threat, through reducing new infections and deaths by 90 and 65%, respectively, by 2030 [1]. To achieve this, in 2016, the World Health Organization (WHO) launched a global strategy which aimed to diagnose 90% of people with HCV chronic infection and treat 80% of eligible patients by 2030 [2].

To monitor progress towards the WHO’s goal, evaluating the cascade of care (CoC) – a widely used tool for HIV infection [3] – may also be effective for HCV infection. This consists in estimating the numbers or percentages of infected people at various stages of the care pathway, from diagnosis to treatment. Identifying gaps in the CoC can help guide Public health policy. Although numerous studies have evaluated the HCV CoC using prospective or retrospective cohorts (i.e., a longitudinal approach), their samples comprised captive, often local, populations, including people attending screening or clinical centres [4, 5], which limits extrapolation of their results to the wider population. Evaluating the HCV CoC at a national level is essential, but challenging. It is often based on modelled data [6, 7] or on combining existing national data sometimes covering a large period of time [8–10]. However, the latter approach is not adapted to the rapidly evolving situation in the era of DAAs. Furthermore, published studies on the HCV CoC in the DAA era are scarce [7, 11, 12].

France has been involved for many years in the fight against hepatitis C, in particular through three national plans implemented since the end of the 1990s [13]. This has led to a slightly more favourable epidemiological situation than in most countries [14, 15], with an estimated viremic infection prevalence in mainland France in 2004 of 0.53% (95% Confidence Interval (CI): 0.40–0.70), corresponding to 232,000 chronically-infected individuals (95% CI: 167,869–296,523) in the 18–80 years old (y.o.) general population, and an estimated 57% (95% CI: 41–71) of these people being aware of their infection, corresponding to 132,000 (95% CI: 95,000-165,000) individuals [16]. Since then, HCV screening activity has continuously increased, reaching 62 anti-HCV tests per 1000 inhabitants in 2016 (vs. 53 / 1000 in 2010) [17], despite no change in official screening recommendations for targeted people at risk of exposure. With regard to antiviral treatment, 1st and 2nd-wave DAAs have been rapidly and widely implemented in France, since 2011 and 2014, respectively, despite their restricted access for people with severe liver disease until mid-2016 [18]. In this favourable context, France has committed itself to achieving HCV elimination by 2025 [19].

National evaluations of the CoC for a given year are needed to monitor progress towards HCV elimination and to assess the impact of DAAs. Accordingly, in this study, we aimed to evaluate the CoC for chronic HCV in mainland France in 2011 and in 2016, before and after the introduction of 2nd-wave DAAs.

## Methods

We used a cross-sectional approach to estimate chronic HCV CoC in 2011 and in 2016.

### Stages of the HCV cascade of care

For both study years, we estimated the numbers of people 1) with chronic HCV infection, 2) aware of their infection, 3) receiving care for chronic HCV infection during the year, and 4) on antiviral treatment during the year. These numbers were estimated for the adult general population in mainland France aged 18–80 years for 2011, and 18–75 years for 2016 in the main analysis (the difference in age range being due to data sources, see below). A complementary analysis, incorporating age range adjustment, compared 18–80 years for 2016 with those from 2011.

The main data sources are summarized in Table 1 and presented below and in [20].

**Table 1:** Description of methodologies used for the estimation of the chronic HCV cascade of care in 2011 and 2016 in mainland France

Estimation of the numbers of people	2011	2016
With chronic HCV infection	Modelling using the multi-parameter evidence synthesis method	Cross-sectional survey (BaroTest)
Aware of their infection	Projection modelling	Cross-sectional survey (BaroTest)
Receiving care for chronic HCV infection during the year	National Health Data System analysis	National Health Data System analysis
On antiviral treatment during the year	National Health Data System analysis	National Health Data System analysis

### Estimation of the numbers of people with chronic HCV infection and people aware of their infection

#### For 2011

**Fig. 1:**
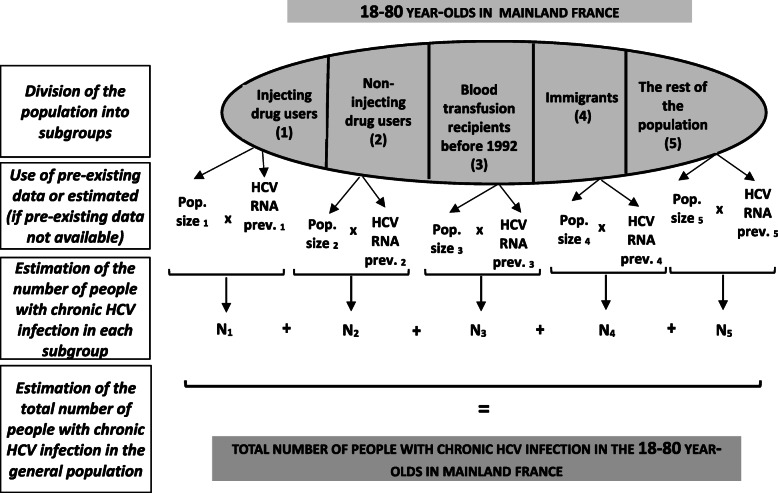
Schematic overview of the principle of the multi-parameter evidence synthesis method in 2011. Pop. = population; prev. = prevalence

The number of people with HCV chronic infection was estimated using the multi-parameter evidence synthesis method (MPES) [21]. This work, published elsewhere [22], consisted in dividing the whole population aged 18–80 years into five subgroups: injecting and non-injecting drug users, transfusion recipients before 1992, immigrants and the rest of the population (Fig. 1). For each subgroup, population size and HCV prevalence (HCV antibodies and HCV RNA) - determined from pre-existing estimates or estimated using available data - were used to obtain the number of infected people (Additional file 1). The five different numbers were combined to estimate the total number of people with HCV chronic infection and the prevalence among the adult population in mainland France. A Bayesian model was used to pool these different data and their uncertainties. The distribution of the number of chronically infected people was estimated using Monte-Carlo simulations (*n* = 80,000) and summarized using the median and 95% credible interval (CrI).

**Fig. 2:**
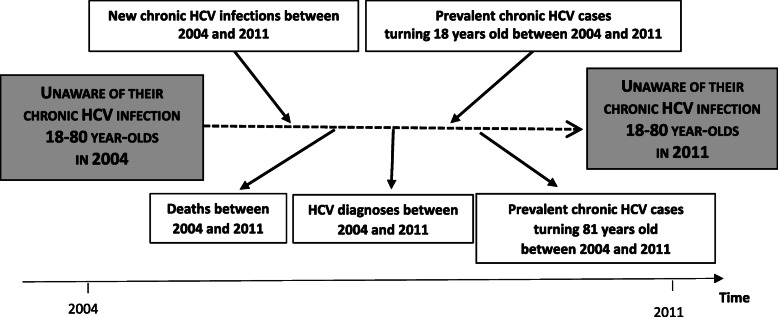
Schematic overview of the projection modelling used to estimate the number of people unaware of their infection in 2011. Each year, individuals enter or leave the pool of the chronically-infected undiagnosed population

The number of people aware of their infection was obtained by subtracting the estimated number of people unaware of their infection from the estimated number of chronically infected people in 2011. The former number was estimated using projection modelling (described elsewhere [23]) from the 2004 national prevalence survey, a cross-sectional survey with biological samples and face-to-face interviews, in particular on HCV tests history [16]. Briefly, to estimate the number of people unaware of their infection in 2011, the epidemiologic model made projections from the estimated number of people unaware of their infection in 2004 (for each year, gender and age-group), taking into account mortality, HCV incidence and diagnosis rates (Fig. 2). Model parameters are described in Additional file 2. To include uncertainties of the model parameters, several scenarios were studied to estimate a plausible interval around the final estimate of the number of people unaware of their chronic infection.

#### For 2016

The numbers of people with chronic HCV infection and people aware of their infection were both estimated using the national cross-sectional 2016-Health Barometer (2016-HB), which is a telephone-based survey conducted in 2016 on a large random sample (*n* = 20,032) of people from the general population in mainland France. A virological sub-study called BaroTest was nested in 2016-HB, and was conducted among participants aged 18–75. BaroTest used home-based blood self-sampling on dried blood spots (DBS). From these DBS, detection of HCV antibodies and HCV RNA (in case of anti-HCV-positive samples) was performed [24, 25].

The number of people with HCV chronic infection was estimated by extrapolating the estimated HCV RNA prevalence to all 18–75 y.o. in the general population in mainland France, using the 2016 estimated population figure from the French Institute for Statistics and Economic Studies (Insee) [26].

The proportion of people with chronic HCV infection aware of their status was defined as the proportion of people among those tested positive for HCV RNA in the BaroTest study, who reported - during the 2016-HB telephone interview - that they had been previously screened for HCV, and that the result of their last HCV test was positive. This proportion was then applied to the 2016 estimated population figure for 18–75 y.o. persons living in mainland France (Insee) [26] in order to estimate the number of people with HCV chronic infection aware of their infection.

More details on these estimates are provided in [24].

### Estimation of the numbers of people receiving care for chronic HCV infection and people on HCV antiviral treatment

For 2011 and 2016, these estimates were performed using data from the French National Health Data System (SNDS) for all France’s health insurance schemes. This health administrative database covers almost the entire French population [27, 28]. It contains individual data on all outpatient healthcare reimbursements (including drugs, biological tests, medical procedures), on hospitalizations in public and private hospitals (in particular admission and discharge dates, primary, related and associated diagnoses) and demographical data. All drugs dispensed in retail pharmacies (and reimbursed) and those dispensed in public and private hospitals (if they are on the list of expensive drugs, such as DAAs) are included. The SNDS also includes information on long-term diseases (LTD). Persons with LTD status in France are fully reimbursed for related healthcare expenditures. However, it does not contain any clinical information about the context of medical consultations, of drug prescriptions or examination results, including biological test results.

To identify people receiving care for chronic HCV infection using the SNDS, an algorithm was constructed in collaboration with clinical and technical experts on health administrative databases. Criteria were defined to identify patients at different stages of the care pathway (initial assessment, treatment etc.). Four successive versions of the algorithm were developed and their results were discussed with the two expert groups before the final algorithm (named “principal algorithm” hereafter) was implemented. We split criteria types into “major” (a high presumed specificity with regard to chronic hepatitis C), “intermediate” (intermediate presumed specificity), and “minor” (low presumed specificity). Care for chronic HCV infection was defined as meeting either at least one major criterion or both the one designated intermediate criterion and at least one minor criterion. Because it was essential to identify people receiving care for chronic HCV infection, excluding people with resolved infection, we chose a very specific definition for the principal algorithm. To assess the impact of this specific definition on the results, an “alternative algorithm”, which was more sensitive, was constructed. These algorithms are presented in Table 2, and details about the various codes used for them are provided in Additional file 3.

**Table 2:** Algorithms (principal and alternative) to identify people receiving care for chronic HCV infection from the French National Health Data System

		Principal algorithm	***Alternative algorithm***
Items		Definitions	***Modifications compared with principal algorithm***
**AT LEAST ONE MAJOR CRITERION DURING THE YEAR AMONG THE FOLLOWING:**			
	HCV genotyping	≥ 1 reimbursement	
	HCV antiviral treatment	≥ 1 reimbursement for pegylated interferon and ribavirin (with at least one common delivery date) or for first or second generation DAAs	
	HCV RNA quantitative PCR	≥ 3 reimbursements	*≥ 2*
	Hospitalization with a principal or related diagnosis of chronic hepatitis C	≥ 1 hospitalization with a principal or related diagnosis of chronic hepatitis C in medicine, surgery or obstetric services	
	Long-term disease (LTD) registration for chronic hepatitis C (“incident patients”)	New LTD registrations for chronic hepatitis C	
**OR**			
**BOTH THE INTERMEDIATE CRITERION DURING THE YEAR:**			
	HCV RNA quantitative PCR	2 reimbursements	*1*
**AND AT LEAST ONE MINOR CRITERION DURING THE YEAR AMONG THE FOLLOWING:**			*≥ 2*
	Liver fibrosis assessment	≥ 1 reimbursement for liver biopsy, fibrosis, liver stiffness measurement or blood biomarkers	
	Long-term disease registration for chronic hepatitis C (“prevalent patients”)	Patients registered with LTD status, excluding those admitted during the year	
	Hospitalization with an associated diagnosis of chronic hepatitis C	≥ 1 hospitalization with an associated diagnosis of chronic hepatitis C in medicine, surgery or obstetric services	

Details of selected codes are provided in Additional file 3

To estimate the number of persons on HCV antiviral treatment in 2011 and in 2016, people who had at least one reimbursement for pegylated interferon and ribavirin (with at least one common delivery date) or at least one reimbursement for first or second generation DAAs, were identified in the SNDS. Details are provided in [18] and in Additional file 3.

### Analysis with age range adjustment

In the main analysis, the estimation of chronic HCV CoC concerned 18–80 y.o. for 2011 and 18–75 y.o. for 2016, because of the data sources available to estimate the numbers of people with chronic HCV infection and people aware of their infection. To assess the impact of this difference in study age range on the evolution of the CoC between the two study years, the CoC was estimated for the 18–80 y.o. for 2016 in a complementary analysis which adjusted for the age range difference.

The additional number of persons with chronic HCV infection in the 76–80 y.o. in 2016 was estimated by applying the prevalence estimated in the 65–75 y.o. in the BaroTest study [24, 25] to the estimated population of 76–80 y.o. persons living in mainland France in 2016 (Insee) [26]. The total estimated number of persons with chronic HCV infection in the 18–80 y.o. in 2016 was then applied to the proportion of persons aware of their infection among the 18–75 y.o. estimated from the BaroTest, in order to calculate the total number of persons aware of their infection among 18–80 y.o. persons.

The numbers of people aged 18–80 receiving care and on antiviral treatment in 2016 were estimated from the analysis of SNDS data.

## Results

**Fig. 3:**
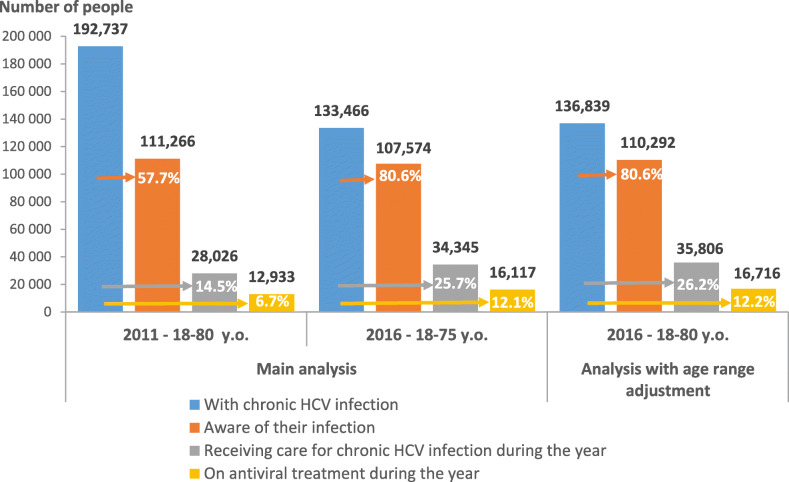
Estimated chronic HCV cascade of care in 2011 and in 2016 in mainland France according to main analysis and analysis with age range adjustment. Note: Percentages are calculated from the estimated numbers of people with chronic HCV infection

The estimated numbers of people at the different stages of the chronic HCV CoC according to the main and age range-adjusted analyses, and their evolution between 2011 and 2016, are presented in Table 3. The corresponding CoC for both years are illustrated in Fig. 3.

**Table 3:** Estimates of the numbers of persons at the different stages of the chronic HCV cascade of care in 2011 and 2016 in mainland France (main analysis and analysis with age range adjustment)

Estimated numbers of people	Main analysis			Analysis with age range adjustment	
	2011 18–80 years	2016 18–75 years	Evolution 2011 / 2016	2016 18–80 years	Evolution 2011 / 2016
With chronic HCV infection	**192,737** (95% CrI: 150,935-246,055)	**133,466** (95% CI: 56,880-312,616)	**−30.8%**	**136,839**	**−29.0%**
Aware of their infection	**111,266** (plausible interval: 76,050-158,000)	**107,574** (95% CI: 58,992-127,594)	**−3.3%**	**110,292** (95% CI: 60,483-130,818)	**−0.9%**
Receiving care for chronic HCV infection during the year	**28,026** (alternative algorithm: 31,371)	**34,345** (alternative algorithm: 41,095)	**+ 22.5%**	**35,806** (alternative algorithm: 42,950)	**+ 27.8%**
On antiviral treatment during the year	**12,933**	**16,117**	**+ 24.6%**	**16,716**	**+ 29.3%**

*CrI* Credible interval, *CI* Confidence interval

### Main analysis

Between 2011 and 2016, chronic HCV infection prevalence decreased from 0.42% (95%CrI: 0.33–0.53) to 0.30% (95%CI: 0.13–0.70). The number of people with chronic HCV infection decreased by 30.8%, from 192,737 to 133,466. While the number of those aware of their infection remained stable (111,266 in 2011, 107,574 in 2016), the relative proportion of this sub-group rose from 57.7 to 80.6%.

In the same period, the numbers of people receiving care for chronic HCV infection and on antiviral treatment increased by 22.5% (from 28,026 to 34,345) and 24.6% (from 12,933 to 16,117), respectively. Between 2011 and 2016, their proportion among the people with chronic HCV infection increased from 14.5 to 25.7% and from 6.7 to 12.1%, respectively. Among people aware of their infection, 25.2% were receiving care for HCV chronic infection and 11.6% were on antiviral treatment in 2011, versus 31.9 and 15.0% in 2016, respectively.

The alternative algorithm, which was more sensitive, identified 31,371 people in care for chronic HCV infection in 2011 and 41,905 in 2016 (i.e., 11.9 and 19.7% additional persons, respectively, compared with the principal algorithm).

### Analysis with age range adjustment

Taking into account the 76–80 y.o. in the 2016 CoC estimation led to an increase of 2.5% in both the number of people with chronic HCV infection and in people aware of their infection, 4.3% in the number of people receiving care, and 3.7% in the number of people on treatment.

In the age range-adjusted analysis, between 2011 and 2016, the decreases in the numbers of infected people and people aware of their infection were slightly lower than in the main analysis, while the increases in the numbers of people receiving care and people on treatment were higher (by approximately five percentage points) compared with the main analysis (Table 3).

Including the 76–80 y.o. in the 2016 HCV CoC had little impact on the proportions of infected people at the different CoC stages compared with the main analysis (Fig. 3).

## Discussion

This work provides the first estimate of the French national CoC for chronic hepatitis C for 2 years, before and after the introduction of 2nd-wave DAAs, in the context of the goal to eliminate HCV. Between 2011 and 2016, the number of people with HCV chronic infection decreased by 30%, the proportion of people aware of their infection substantially increased (from 57.7 to 80.6%), and the numbers of people receiving care for HCV infection and on antiviral treatment during the year both increased by one quarter.

This work has several strengths: first, it focused on the French population at the national level (i.e., not local or specific populations), enabling us to assess French progress toward the WHO’s elimination objective [2]. Second, given the rapidly evolving situation in recent years, with the advent of DAAs in recent years, it provided estimates of the different stages of the CoC related to the same year (whether 2011 or 2016), not a larger period [8–10]. Third, almost all estimates of the numbers of people at the different stages were independently calculated. Estimates in some previous studies of national CoC [8, 10, 29] were interdependent, which could pose problems in the event of an error in one of the estimates. Fourth, SNDS data are not subject to selection bias because they cover almost the entire French population. Finally, by estimating the HCV CoC before (2011) and after (2016) the introduction of 2nd-wave DAAs, we were able to assess these drugs’ initial impact on the CoC.

Our work also has limitations: first, different methodologies were used to estimate the numbers of people with chronic HCV infection and those aware of their infection, specifically, modelling studies for 2011 [22, 23] and a cross-sectional survey (BaroTest) in the general population for 2016 [24]. In the latter type of survey, some populations at risk of HCV (e.g., active injecting drug users) are frequently not represented or are underrepresented, leading to possible underestimation of national prevalence. The difference we observed between 2011 and 2016 prevalence estimates is therefore probably overestimated. Second, given the low prevalence in the general population, the number of persons testing positive in BaroTest was small, leading to poor robustness in the estimated proportion of people aware of their infection in 2016 [24]. Third, because SNDS does not include biological test results or data on consultation diagnoses, we had to construct algorithms to identify people receiving HCV care. Validation studies would be necessary to assess these algorithms’ performance. Fourth, the identification of people on antiviral treatment depends on coding quality, which was suboptimal for DAAs in 2014–2015 but less so in 2016 [18, 30]. Finally, because of data sources, our CoC could not consider people older than 80 y.o. or those living in French overseas areas and could not be presented according to age, gender, at-risk exposures and time from infection (to focus on new or re-infected people).

The decrease in chronic hepatitis C prevalence (from 0.42% in 2011 to 0.30% in 2016) is probably mostly linked to highly effective 2nd-wave DAAs which led to at least 20,000 people in France being cured in 2014–2015 [18]. A decrease in prevalence (from 1 to 0.6%) was also observed in a population-level CoC in British Columbia between 2012 and 2018 [11]. This trend can also be explained by deaths and aging in the HCV population beyond the age limit of the prevalence surveys (80 years for 2011, 75 years for 2016). Indeed, in France, a large percentage of HCV contaminations occurred before the implementation of systematic testing of blood donors and harm reduction measures in the 1990s, leading to a rather elderly population of people with chronic HCV infection. A continuation of the decrease in the incidence among people who actively inject drugs - observed between 2004 and 2011 (from 15.4 to 11.2 per 100 person-years) [31] - is another possible explanation.

The increasing trend in the proportion of chronically infected people aware of their infection observed in our study is coherent with other published estimates for France [6, 15, 32]. This trend may be partly explained by the substantial increase in anti-HCV screening activity in laboratories (+ 21% between 2010 and 2016) [17], which in turn is possibly related to the availability of DAAs and to experts’ recommendations in 2014 and 2016 advocating universal screening [14, 33]. In addition, the decrease in the number of elderly infected patients (whether through death or aging beyond the age limit of related studies), which constituted the age-group with the highest proportion of undiagnosed cases [23], may have contributed to the large observed increase in the proportion of people aware of their infection between 2011 and 2016.

The numbers of people receiving care for chronic HCV infection and on antiviral treatment during the year increased by 22.5 and 24.6%, respectively, between 2011 and 2016. As depicted in Additional file 4, these numbers evolved in parallel with the advent of new antiviral drugs.

Our results show a substantial improvement in all the stages of the chronic HCV CoC over a short 5-year period, characterized by major therapeutic innovations, specifically the advent of 1st and 2nd-wave DAAs, which were rapidly and widely implemented in France [18].

In 2016, according to our estimates, among people with chronic HCV infection, more than eight in ten were aware of their infection, one in four received care for their HCV infection, and one in eight were on antiviral treatment during the year. Our results are relatively consistent with 2015 Markov model-based estimates by the European Union HCV Collaborators group which found that among chronically-infected people in France (estimated prevalence = 0.29%), 74.1% were diagnosed and 10.2% started treatment [6]. Moreover, the group’s estimates for the European Union as a whole (estimated prevalence = 0.64%) [6], were 36.7 and 7.5%, respectively, which highlights France’s favourable situation when compared with other countries in the European Union (4th position for the diagnosis rate, and 2nd position for the proportion of persons treated) [6], which is even more favourable when compared with the rest of the world (20% - diagnosis and 1.5% - newly treated) [34]. Since 2016, these figures have placed France on the short list of countries considered to be on track to eliminate hepatitis C by 2030 [35]. A recent modelling study projected that France will reach HCV elimination by 2024 [36]. Indeed, France has already met the eight criteria identified in the study for successful HCV elimination, in particular the following essential criteria: i) political will, ii) a financed “priority prevention” plan (in 2018 as a result of criterion i)) which aimed to achieve HCV elimination by 2025 [19], iii) the removal of treatment restrictions in mid-2016 (which led to a 35% increase in the number of patients initiating a DAA treatment between 2016 and 2017 [30]), and iv) monitoring and evaluation of existing programs. The other criteria are: v) the expansion of treatment provision beyond specialists (DAAs can be delivered in retails pharmacies since March 2017 and prescribed by all physicians since May 2019), vi) the implementation of an awareness campaign in the general population (“Noise against the Hepatitis C”) [37], vii) a national screening program (annual local campaigns since 2019 and a national linkage to care program with outreach actions), and viii) the existence of harm-reduction programs (in particular, reinforced test and treat programs) [38].

Although it is expected that this comprehensive elimination program implemented in France in recent years will help enhance the country’s HCV CoC, our results demonstrate that in 2016, France was still far from reaching WHO’s elimination targets, in particular regarding the proportion of treated patients (only 12% versus the WHO objective of 80%) [2]. Our work highlights that care management for chronic HCV infection constitutes a major gap with an estimated 31.9% people receiving care among those aware of their infection in 2016. The choice of a conservative algorithm to identify people in care may partly explain this low proportion, which should be considered as a minimum. However, the application of the alternative, more sensitive algorithm led to a slightly increased proportion (38.2%). Universal access to DAAs in France (announced in mid-2016 but only effective since mid-2017), may further increase this proportion. The diagnosis of infected people also constitutes a challenge for HCV elimination. Experts and patient associations strongly advocated universal screening of all adults at least once during their lifetime, in addition to risk-based targeted testing [14, 33], after a modelling study showed that this new strategy could be cost-effective [39]. However, in 2019, the French National Authority for Health concluded that its effectiveness was not sufficiently demonstrated [40].

## Conclusion

Our study demonstrates a marked improvement in the CoC for chronic HCV infection between 2011 and 2016, suggesting the substantial impact of 2nd-wave DAAs in mainland France. However, access to care and antiviral treatment remained insufficient in 2016, when considering the WHO’s elimination target of 2030. These national estimates will have to be updated to assess the impact of new measures implemented since 2016, and to monitor the elimination of HCV by 2025, as planned by France. Further studies are also needed to estimate the CoC among specific populations including people who inject drugs.

## Supplementary information

**Additional file 1: Table S1.** Data sources for the estimation of the number of people with HCV chronic infection in 2011 in mainland France

**Additional file 2: Table S2.** Data sources for the estimation of the number of people unaware of their HCV chronic infection in 2011 in mainland France

**Additional file 3: Table S3**. Code dictionary of medical conditions, medical procedures, medical biology acts and drugs used in the algorithms to identify patients receiving care for chronic HCV infection

**Additional file 4: Figure S1.** Evolution of the estimated numbers of people (18–75 y.o/18–80 y.o): i) receiving care for chronic HCV infection according to the algorithm used (principal/alternative) (A), and ii) on antiviral treatment between 2011 and 2016 (B)

## Data Availability

All data generated or analysed during this study are included in this published article and its supplementary files.

## Abbreviations

CI: Confidence Interval
CrI: Credible interval
CoC: Cascade of care
DAAs: Direct-acting antivirals
DBS: Dried blood spots
HB: Health Barometer
HCV: Hepatitis C Virus
Insee: French Institute for Statistics and Economic Studies
LTD: Long-term diseases
MPES: Multi-parameter evidence synthesis method
PCR: Polymerase chain reaction
RNA: Ribonucleic acid
SNDS: French National Health Data System
WHO: World Health Organization
