# Supplementary material for: French hepatitis C care cascade: substantial impact of direct-acting antivirals, but the road to elimination is still long
Source: BMC Infect Dis. 2020 Oct 15;20:759. doi: 10.1186/s12879-020-05478-6 (PMC7559725; doi:10.1186/s12879-020-05478-6)
Supplement: Supplementary file 1 — Additional file 1: Table S1. Data sources for the estimation of the number of people with HCV chronic infection in 2011 in mainland France [file 12879_2020_5478_MOESM1_ESM.docx]

Table 1: Data sources for the estimation of the number of people with HCV chronic infection in 2011 in mainland France

| **Subgroups** | **Population size** | | **HCV antibodies** | | **HCV RNA** | |
| --- | --- | --- | --- | --- | --- | --- |
|  | **Estimation** | **Source** | **Estimated prevalence (95% confidence interval*)** | **Source** | **Estimated prevalence** | **Source** |
| **Injecting drug users** | 148,000 | French Monitoring Centre for Drugs and Drug Addiction [1] | 63.8%  (59.4-68.3) | ANRS-Coquelicot survey 2011 [2] | 29.6% | Estimated from the proportion of viremic people among individuals with HCV antibodies estimated in ANRS-Coquelicot survey 2011 [2] |
| **Non- injecting drug users** | 132,000 | French Monitoring Centre for Drugs and Drug Addiction [1] | 4.9%  (2.4-7.4) | ANRS-Coquelicot survey 2011 [2] | 2.2% | Estimated from the proportion of viremic people among individuals with HCV antibodies estimated in ANRS-Coquelicot survey 2011 [2] |
| **Blood transfusion recipients before 1992** | 2,831,391 | National HCV prevalence survey 2004 [3] taking into account the evolution of the population between 2004 and 2011 | 3.41% | National HCV prevalence survey 2004 [3] taking into account the evolution of the population between 2004 and 2011 | 2.1% | National HCV prevalence survey 2004 [3] taking into account the evolution of the population between 2004 and 2011 |
| **Immigrants** | 4,938,439 | National Institute of Statistics and Economic Studies (Insee) [4] | 1.83% | Estimated from [5, 6] | 1.0% | Estimated from:  - the HCV antibody prevalence estimated in this subgroup in 2011;  - the proportion of viremic people among individuals with HCV antibodies estimated for the mainland France population from the national HCV prevalence survey 2004 [3] ;  - the evolution of the proportion of viremic people among individuals with HCV antibodies estimated from epidemiological surveillance of blood donors between 2004 and 2011 |
| **Other people** | 38,114,942 | Difference between the size of the population aged 18-80 in mainland France (Insee 2011) and the size of the other population subgroups | 0.15% | Estimated from the national HCV prevalence survey 2004 [3] taking into account the evolution of the HCV prevalence among new blood donors between 2004 and 2011 | 0.09% | Estimated from:  - the HCV antibody prevalence estimated in this subgroup in 2011;  - the proportion of viremic people among individuals with HCV antibodies estimated for the mainland France population from the national HCV prevalence survey 2004 [3] ;  - the evolution of the proportion of viremic people among individuals with HCV antibodies estimated from epidemiological surveillance of blood donors between 2004 and 2011 |

*Source: [7]*

**if appropriate and available*

**References**

[1] Janssen E, Bastianic T. Usage problématique de drogues en France : les prévalences en 2011. Estimations locales et extrapolations nationales. Saint-Denis La Plaine: Observatoire Français des drogues et des toxicomanies; 2013. <https://www.ofdt.fr/publications/collections/rapports/rapports-d-etudes/rapports-detudes-ofdt-parus-en-2013/usage-problematique-de-drogues-en-france-les-prevalences-en-2011-septembre-2013/>

[2] Weill-Barillet L, Pillonel J, Semaille C, Leon L, Le Strat Y, Pascal X, et al. Hepatitis C virus and HIV seroprevalences, sociodemographic characteristics, behaviors and access to syringes among drug users, a comparison of geographical areas in France, ANRS-Coquelicot 2011 survey. Revue d'epidemiologie et de sante publique. 2016;64:301-12.

[3] Meffre C, Le Strat Y, Delarocque-Astagneau E, Dubois F, Antona D, Lemasson JM, et al. Prevalence of hepatitis B and hepatitis C virus infections in France in 2004: social factors are important predictors after adjusting for known risk factors. J Med Virol. 2010;82:546-55.

[4] National Institute of Statistics and Economic Studies (INSEE). <https://www.insee.fr/en/accueil>

[5] Gower E, Estes C, Blach S, Razavi-Shearer K, Razavi H. Global epidemiology and genotype distribution of the hepatitis C virus infection. J Hepatol. 2014;61:S45-57.

[6] Lavanchy D. Evolving epidemiology of hepatitis C virus. Clin Microbiol Infect. 2011;17:107-15.

[7] Pioche C, Pelat C, Larsen C, Desenclos JC, Jauffret Roustide M, Lot F, et al. Estimation de la prévalence de l'hépatite C en population générale, France métropolitaine, 2011. Numéro thématique. Hépatites B et C, données épidémiologiques récentes. Bull Epidemiol Hebd. 2016:224-9.
