# Supplementary material for: French hepatitis C care cascade: substantial impact of direct-acting antivirals, but the road to elimination is still long
Source: BMC Infect Dis. 2020 Oct 15;20:759. doi: 10.1186/s12879-020-05478-6 (PMC7559725; doi:10.1186/s12879-020-05478-6)
Supplement: Supplementary file 2 — Additional file 2: Table S2. Data sources for the estimation of the number of people unaware of their HCV chronic infection in 2011 in mainland France [file 12879_2020_5478_MOESM2_ESM.docx]

***Table 2: Data sources for the estimation of the number of people unaware of their HCV chronic infection in 2011 in mainland France***

| **Parameters** | **Data** | | **Estimates used for modelling** | **References** |
| --- | --- | --- | --- | --- |
| **New HCV infections evolving to chronicity among “active IDUs”** | Estimated number of active IDUs in France in : |  |  |  |
|  |  | 2006 | 81,000 | [1] |
|  |  | 2011 | Low: 70,000; high: 90,000 | Assumed from [2] |
|  | Age-and-gender distribution of French active IDUs in: |  |  |  |
|  |  | 2004 |  | [3] |
|  |  | 2011 |  | [4] |
|  | HCV seroprevalence among active IDUs in France, by age-group and gender in: |  |  |  |
|  |  | 2004 |  | [3] |
|  |  | 2011 |  | [4] |
|  | HCV incidence in IDUs in western countries |  | Low: 6%, high: 18% | [5-15] |
|  | Rate of HCV spontaneous clearance |  | Low: 30%, high: 40% | [16, 17] |
| **New HCV infections evolving to chronicity among the “general population”** | Annual HCV incidence in repeat blood donors by age-group and gender in France. |  |  | Method in [18] |
|  | Distribution of the French general population by age-group and gender. |  |  | [19] |
|  | HCV seroprevalence in the French general population by age-group and gender. |  |  | [20] |
|  | Rate of HCV spontaneous clearance |  | Low: 30%, high: 40% | [16, 17] |
| **Prevalent undiagnosed cases turning 18 between 2004 and 2011** | Number of chronically-infected undiagnosed HCV cases aged 18 by gender in 2004 in France |  | Men: 112, women: 122 | [20] |
| **Number of diagnoses among prevalent HCV cases in general population in 2004** | Estimated proportion of chronically-infected HCV cases aware of their status in 2004 by age-group and gender. |  |  | [20] |
|  | Assumption of an annual increase of 1% in the proportion of HCV cases aware of their status |  |  | [21] |
| **Number of diagnoses among new HCV infections cases in general population between 2004 and 2011** | Estimated proportion of chronically-infected HCV cases aware of their status in 2004 among French general population. |  |  | [20] |
|  | Distribution of the delay between at-risk exposure period and diagnosis of HCV cases for whom intravenous drug use and blood transfusion were not suspected |  | 0-4 years: 20%, 5-10 years: 15%, > 10: 65% | [22] |
| **Number of diagnoses among new HCV infections cases in active IDUs between 2004 and 2011** | Estimated proportion of anti-HCV IDUs aware of their status in: |  |  |  |
|  |  | 2004 | 69% | [3] |
|  |  | 2011 | 78% | [4] |
|  | Age at first injection in: |  |  |  |
|  |  | 2004 | 20.4 | [3] |
|  |  | 2011 | 22 | [4] |
|  | Distribution of the delay between at-risk exposure period and diagnosis of HCV cases for whom intravenous drug use was the suspected transmission mode |  | 0-4 years: 47%, 5-10 years: 33%, > 10: 20% | [22] |
| **Number of deaths** | Competitive mortality based on French life tables |  |  | [19] |
|  | Additional mortality for active IDU incident cases between 2004 and 2011 |  | 5.27 for men, 9.74 for women | [23] |
| **Undiagnosed HCV cases turning 81 between 2004 and 2011** | Exclusion of estimated undiagnosed cases aged over 80 in 2011 |  | From 2011 model-based estimates |  |

*IDUs: Intravenous drug users*

*Source: [24]*

***References***

[1] Costes JM. Prévalence de l'usage problématique de drogues en France : estimations 2006. Tendances. 2009:1-4. <https://www.ofdt.fr/BDD/publications/docs/eftxjcpc.pdf>

[2] Janssen E, Bastianic T. Usage problématique de drogues en France : les prévalences en 2011. Estimations locales et extrapolations nationales. Saint-Denis La Plaine: Observatoire Français des drogues et des toxicomanies; 2013. <https://www.ofdt.fr/publications/collections/rapports/rapports-d-etudes/rapports-detudes-ofdt-parus-en-2013/usage-problematique-de-drogues-en-france-les-prevalences-en-2011-septembre-2013/>

[3] Jauffret-Roustide M, Le Strat Y, Couturier E, Thierry D, Rondy M, Quaglia M, et al. A national cross-sectional study among drug-users in France: epidemiology of HCV and highlight on practical and statistical aspects of the design. BMC infectious diseases. 2009;9:113.

[4] Jauffret-Roustide M, Pillonel J, Weill-Barillet L, Leon L, Le Strat Y, Brunet S, et al. Estimation de la séroprévalence du VIH et de l'hépatite C chez les usagers de drogues en France - Premiers résultats de l'enquête ANRS-Coquelicot 2011. BEH N°39-40. 2013.

[5] van den Berg CH, Smit C, Bakker M, Geskus RB, Berkhout B, Jurriaans S, et al. Major decline of hepatitis C virus incidence rate over two decades in a cohort of drug users. European journal of epidemiology. 2007;22:183-93.

[6] Brant LJ, Ramsay ME, Balogun MA, Boxall E, Hale A, Hurrelle M, et al. Diagnosis of acute hepatitis C virus infection and estimated incidence in low- and high-risk English populations. J Viral Hepat. 2008;15:871-7.

[7] Hope VD, Hickman M, Ngui SL, Jones S, Telfer M, Bizzarri M, et al. Measuring the incidence, prevalence and genetic relatedness of hepatitis C infections among a community recruited sample of injecting drug users, using dried blood spots. J Viral Hepat. 2011;18:262-70.

[8] Craine N, Hickman M, Parry JV, Smith J, Walker AM, Russell D, et al. Incidence of hepatitis C in drug injectors: the role of homelessness, opiate substitution treatment, equipment sharing, and community size. Epidemiology and infection. 2009;137:1255-65.

[9] Hagan H, Latka MH, Campbell JV, Golub ET, Garfein RS, Thomas DA, et al. Eligibility for treatment of hepatitis C virus infection among young injection drug users in 3 US cities. Clin Infect Dis. 2006;42:669-72.

[10] Grebely J, Lima VD, Marshall BD, Milloy MJ, DeBeck K, Montaner J, et al. Declining incidence of hepatitis C virus infection among people who inject drugs in a Canadian setting, 1996-2012. PLoS One. 2014;9:e97726.

[11] Grebely J, Matthews GV, Lloyd AR, Dore GJ. Elimination of hepatitis C virus infection among people who inject drugs through treatment as prevention: feasibility and future requirements. Clin Infect Dis. 2013;57:1014-20.

[12] Wandeler G, Gsponer T, Bregenzer A, Gunthard HF, Clerc O, Calmy A, et al. Hepatitis C virus infections in the Swiss HIV Cohort Study: a rapidly evolving epidemic. Clin Infect Dis. 2012;55:1408-16.

[13] Page-Shafer K, Pappalardo BL, Tobler LH, Phelps BH, Edlin BR, Moss AR, et al. Testing strategy to identify cases of acute hepatitis C virus (HCV) infection and to project HCV incidence rates. Journal of clinical microbiology. 2008;46:499-506.

[14] Lucidarme D, Bruandet A, Ilef D, Harbonnier J, Jacob C, Decoster A, et al. Incidence and risk factors of HCV and HIV infections in a cohort of intravenous drug users in the North and East of France. Epidemiology and infection. 2004;132:699-708.

[15] Valdiserri R, Khalsa J, Dan C, Holmberg S, Zibbell J, Holtzman D, et al. Confronting the emerging epidemic of HCV infection among young injection drug users. Am J Public Health. 2014;104:816-21.

[16] Amin J, Law MG, Micallef J, Jauncey M, Van Beek I, Kaldor JM, et al. Potential biases in estimates of hepatitis C RNA clearance in newly acquired hepatitis C infection among a cohort of injecting drug users. Epidemiology and infection. 2007;135:144-50.

[17] Laperche S, Servant Delmas A, Gallian P, Pillonel J. La surveillance de la diversité des virus VIH, VHB et VHC chez les donneurs de sang français entre 2000 et 2010. Numéro thématique. Don de sang : surveillance du risque infectieux et sécurité transfusionnelle. Bull Epidemiol Hebd. 2012:447-52.

[18] Pillonel J, Laperche S. Trends in risk of transfusion-transmitted viral infections (HIV, HCV, HBV) in France between 1992 and 2003 and impact of nucleic acid testing (NAT). Euro surveillance : bulletin Europeen sur les maladies transmissibles = European communicable disease bulletin. 2005;10:5-8.

[19] Institut national de la statistique et des études économiques - <http://www.insee.fr/fr/bases-de-donnees>. 2011.

[20] Meffre C, Le Strat Y, Delarocque-Astagneau E, Dubois F, Antona D, Lemasson JM, et al. Prevalence of hepatitis B and hepatitis C virus infections in France in 2004: social factors are important predictors after adjusting for known risk factors. J Med Virol. 2010;82:546-55.

[21] Deuffic-Burban S, Deltenre P, Buti M, Stroffolini T, Parkes J, Muhlberger N, et al. Predicted effects of treatment for HCV infection vary among European countries. Gastroenterology. 2012;143:974-85.e14.

[22] Delarocque-Astagneau E, Meffre C, Dubois F, Pioche C, Le Strat Y, Roudot-Thoraval F, et al. The impact of the prevention programme of hepatitis C over more than a decade: the French experience. J Viral Hepat. 2010;17:435-43.

[23] Lopez D, Martineau H, Palle C. Mortalité liée aux drogues illicites. Etude de cohorte rétrospective de personnes interpellées pour usage de stupéfiants. Saint-Denis La Plaine: Observatoire français des drogues et des toxicomanies; 2004. <https://www.ofdt.fr/BDD/publications/docs/epfxdlk7.pdf>

[24] Brouard C, Le Strat Y, Larsen C, Jauffret-Roustide M, Lot F, Pillonel J. The undiagnosed chronically-infected HCV population in France. Implications for expanded testing recommendations in 2014. PLoS One. 2015;10:e0126920.
