# Supplementary material for: French hepatitis C care cascade: substantial impact of direct-acting antivirals, but the road to elimination is still long
Source: BMC Infect Dis. 2020 Oct 15;20:759. doi: 10.1186/s12879-020-05478-6 (PMC7559725; doi:10.1186/s12879-020-05478-6)
Supplement: Supplementary file 3 — Additional file 3: Table S3. Code dictionary of medical conditions, medical procedures, medical biology acts and drugs used in the algorithms to identify patients receiving care for chronic HCV infection [file 12879_2020_5478_MOESM3_ESM.docx]

***Table 3: Code dictionary of medical conditions, medical procedures, medical biology acts and drugs used in the algorithms to identify patients receiving care for chronic HCV infection***

|  | **International classification of diseases and related health problems, 10^th^ revision, French version (ICD-10-FR)** | **Medical common procedure coding system, French version (CCAM)** | **Medical biology acts coding system, French version (NABM)l** | **Anatomical Therapeutic Chemical (ATC) classification** |
| --- | --- | --- | --- | --- |
| Chronic HCV infection | B18.2 |  |  |  |
| Liver biopsy |  | HLHB001; HLHH001; HLHH005; HLHJ003 |  |  |
| Fibrosis liver stiffness measurement (Fibroscan®) |  | HLQM002 |  |  |
| Liver fibrosis blood biomarkers |  |  | 1000; 1001; 1002 |  |
| Quantitative HCV RNA PCR |  |  | 4124 |  |
| HCV genotyping |  |  | 4125 |  |
| PEGINTERFERON |  |  |  | L03AB10; L03AB11 |
| RIBAVIRIN |  |  |  | J05AB04; J05AP01 |
| TELAPREVIR |  |  |  | J05AE11; J05AP02 |
| BOCEPREVIR |  |  |  | J05AE12; J05AP03 |
| SIMEPREVIR |  |  |  | J05AE14 |
| SOFOSBUVIR |  |  |  | J05AP08 ; J05AX15 |
| SOFOSBUVIR + LEDIPASVIR |  |  |  | J05AP51 ; J05AX65 |
| SOFOSBUVIR + VELPATASVIR |  |  |  | J05AP55 |
| DACLATASVIR |  |  |  | J05AX14; J05AP07 |
| DASABUVIR |  |  |  | J05AX16; J05AP09 |
| OMBITASVIR, PARITAPREVIR ET RITONAVIR |  |  |  | J05AX67; J05AP53 |
| ELBASVIR ET GRAZOPREVIR |  |  |  | J05AX68; J05AP54 |
