# Supplementary material for: French hepatitis C care cascade: substantial impact of direct-acting antivirals, but the road to elimination is still long
Source: BMC Infect Dis. 2020 Oct 15;20:759. doi: 10.1186/s12879-020-05478-6 (PMC7559725; doi:10.1186/s12879-020-05478-6)

***Fig 1: Evolution of the estimated numbers of people (18-75 y.o/18-80 y.o): i) receiving care for chronic HCV infection according to the algorithm used (principal/alternative) (A), and ii) on antiviral treatment between 2011 and 2016 (B)***

***A***

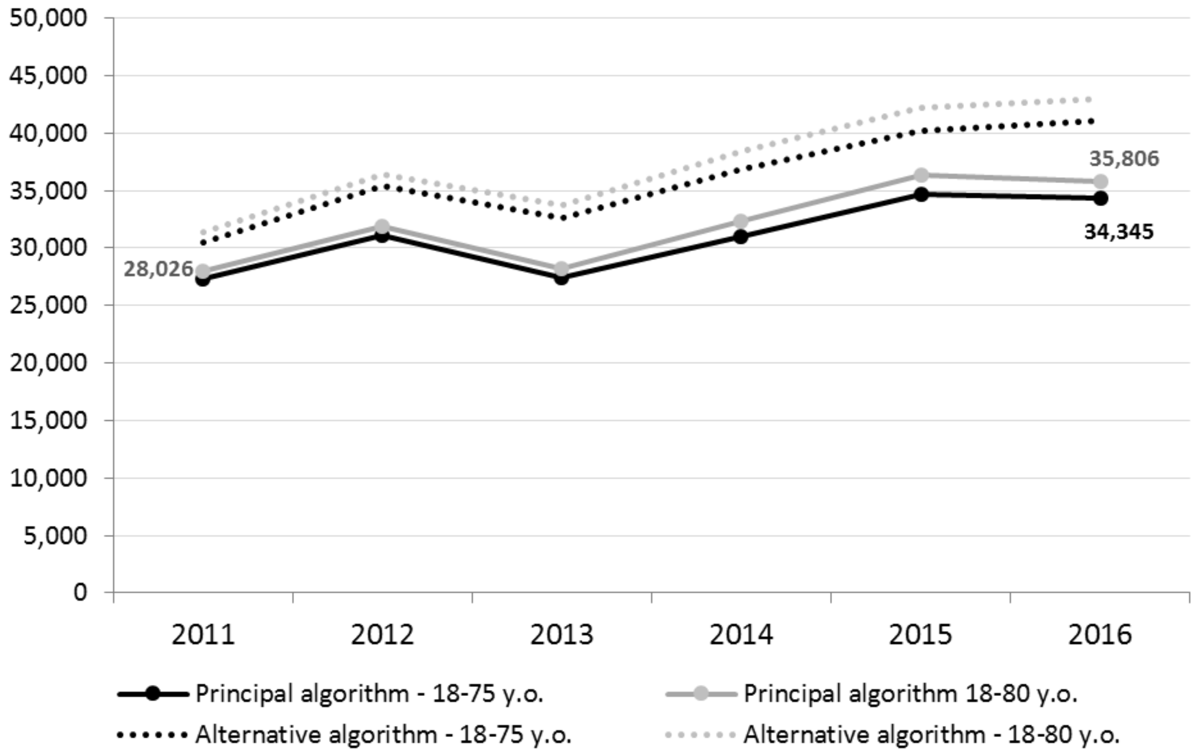


***B***

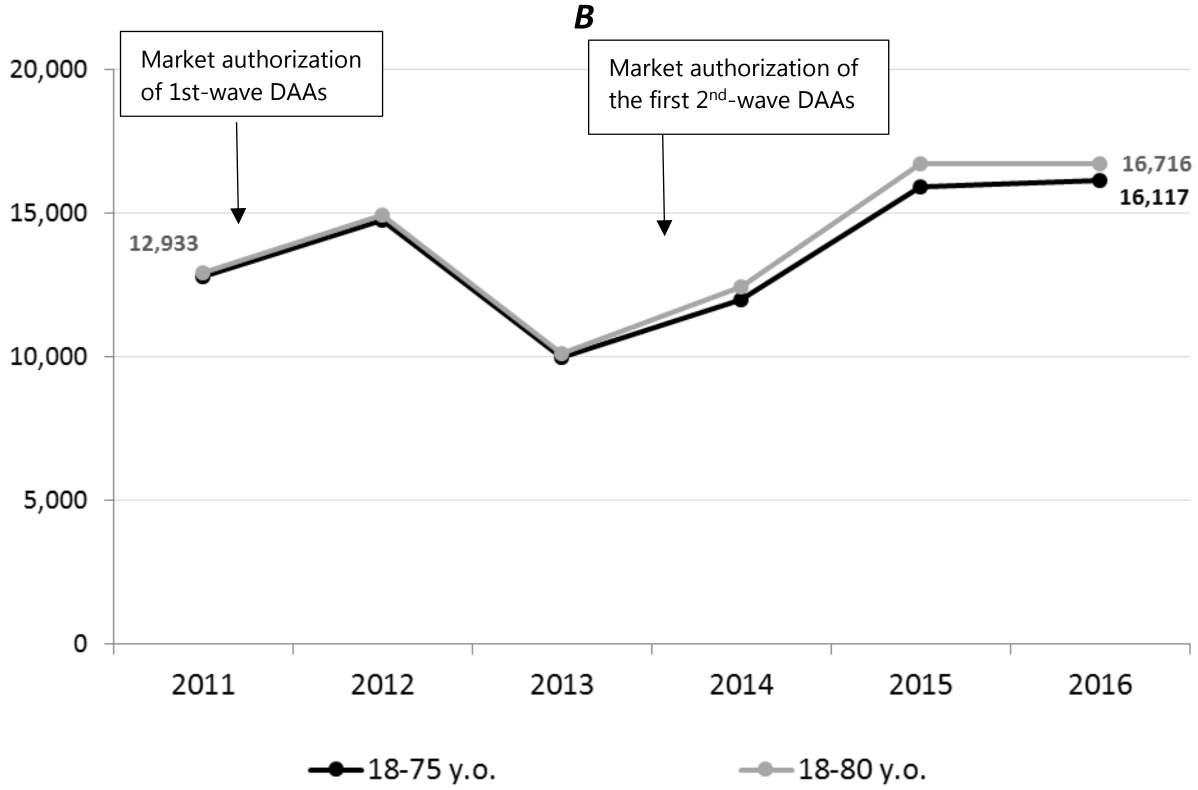

Supplement: Supplementary file 4 — Additional file 4: Figure S1. Evolution of the estimated numbers of people (18–75 y.o/18–80 y.o): i) receiving care for chronic HCV infection according to the algorithm used (principal/alternative) (A), and ii) on antiviral treatment between 2011 and 2016 (B) [file 12879_2020_5478_MOESM4_ESM.pdf]
